# Previous Humoral Immunity to the Endemic Seasonal Alphacoronaviruses NL63 and 229E Is Associated with Worse Clinical Outcome in COVID-19 and Suggests Original Antigenic Sin

**DOI:** 10.3390/life11040298

**Published:** 2021-04-01

**Authors:** Daniele Focosi, Angelo Genoni, Ersilia Lucenteforte, Silvia Tillati, Antonio Tamborini, Pietro Giorgio Spezia, Lorenzo Azzi, Andreina Baj, Fabrizio Maggi

**Affiliations:** 1 North-Western Tuscany Blood Bank, Pisa University Hospital, 56124 Pisa, Italy; 2 Department of Medicine and Surgery, University of Insubria, 21100 Varese, Italy; angelopaolo.genoni@uninsubria.it (A.G.); l.azzi@uninsubria.it (L.A.); andreina.baj@uninsubria.it (A.B.); fabrizio.maggi@uninsubria.it (F.M.); 3 Department of Clinical and Experimental Medicine, University of Pisa, 56100 Pisa, Italy; ersilia.lucenteforte@unipi.it (E.L.); silvia.tillati@med.unipi.it (S.T.); 4 Laboratory of Microbiology, ASST Sette Laghi, 21100 Varese, Italy; antonio.tamborini@asst-settelaghi.it; 5 Department of Translational Research, University of Pisa, 56100 Pisa, Italy; p.spezia@studenti.unipi.it; 6 Unit of Oral Medicine and Pathology, ASST Sette Laghi, 21100 Varese, Italy

**Keywords:** COVID-19, SARS-CoV-2, convalescent plasma, neutralizing antibody, NL63, 229E, OC43, HKU1

## Abstract

Antibody-dependent enhancement (ADE) of severe acute respiratory syndrome coronavirus-2 (SARS CoV-2) infection has been hypothesized. However, to date, there has been no in vitro or in vivo evidence supporting this. Cross-reactivity exists between SARS CoV-2 and other *Coronaviridae* for both cellular and humoral immunity. We show here that IgG against nucleocapsid protein of alphacoronavirus NL63 and 229E correlate with the World Health Organization’s (WHO) clinical severity score ≥ 5 (incidence rate ratios was 1.87 and 1.80, respectively, and 1.94 for the combination). These laboratory findings suggest possible ADE of SARS CoV-2 infection by previous alphacoronavirus immunity.

## 1. Introduction

COVID-19 has totaled more than 100 million cases and more than 2.5 million deaths worldwide as of 17 March 2021. To date, several clinical risk factors for poor COVID-19 outcome have been identified (e.g., age, body mass index (BMI), cardiovascular comorbidities, and humoral immune response), but controversy exists about the role of previous immunity to related coronaviruses. Antibodies against SARS CoV-2 have been shown to correlate with clinical outcome in several large trials, with positivity for anti-spike receptor-binding domain predictive for survival [1]. Theoretically, cross-reacting anti-SARS CoV-2 antibodies could facilitate infection of angiotensin-converting enzyme 2 (ACE-2)-deficient cell types via Fc receptors, and lead to increased viral replication within the body. The phenomenon, called original antigenic sin (OAS) or antibody-dependent enhancement (ADE) of infection, is well known for different viral families (e.g., Dengue virus, Yellow Fever virus, human immunodeficiency virus type 1, respiratory syncytial virus (RSV), Hantavirus, Ebola virus, West Nile virus, etc.) and different coronaviruses, including SARS CoV-2 [2,3]. Different species of alphacoronaviruses and betacoronaviruses infect humans, having variable degrees of similarity and potential cross-reactivity to SARS CoV-2. In silico analysis shows that OC43, HKU1, 229E, and NL63 are expected to induce immune memory against SARS CoV-2 by sharing protein fragments (antigen epitopes) for presentation to the immune system by MHC class I [4]. We thus investigated whether previous immunity to the seasonal (endemic) coronaviruses could affect the clinical outcome of COVID-19.

## 2. Materials and Methods

### 2.1. Patients

In total, 78 consecutive COVID-19 patients (defined as having a SARS CoV-2-positive nasopharyngeal swab with real-time RT-PCR) attending the COVID-19 hospital units were enrolled in the study. The study protocol was approved by the internal review board (protocol number: 165/2020) and all patients provided informed consent. Clinical outcomes were registered according to the highest rank in the World Health Organization’s (WHO) ordinal scale of COVID-19 severity [5]: no limitation of activities (1), limitation of activities (2), hospitalized without oxygen therapy (3), oxygen by mask or nasal prongs (4), non-invasive ventilation or high-flow oxygen (5), intubation and mechanical ventilation (6), ventilation with additional organ support (7), or death (8).

### 2.2. Serology

As per diagnostic protocols, the patients were tested for anti-SARS CoV-2 antibodies using the Liaison S1/S2 IgG assay (DiaSorin, Saluggia, Italy). The residual serum samples were also tested using a recently marketed line immunoassay *recom*Line SARS CoV-2 IgG (Mikrogen Diagnostik GmbH, Neuried, Germany). The strip test simultaneously detects IgG against nucleoproteins (NP) of seasonal coronaviruses 229E, NL63, OC43, HKU1, and to 3 different antigens of SARS-CoV-2 (nucleoprotein, S1 Spike subunit, and receptor-binding domain, RBD). Intensity of the detected antigen bands were determined by automated reading.

### 2.3. Statistical Analysis

Standard statistical methods were used to describe patients’ demographics (age, sex), clinical status (comorbidities and other previous infections), and antibodies against NP of HCoV-229E (NP.229E), HCoV-NL63 (NP.NL63), HCoV-OC43 (NP.OC43), HCoV-HKU1 (NP.HKU1), and 3 different antigens of SARS CoV-2 (NP.SARS.2, RBD.SARS.2, S1.SARS.2).

A frequency analysis was conducted for the considered outcome “maximal WHO score” during the disease course. This dependent variable was defined by the different levels of disease, from score 0 corresponding to an asymptomatic status to score 8, indicating severe diseases.

The partial correlation coefficients were used to measure the relationship between the different independent variables adjusted for sex and age. In particular, we focused our analysis on the nucleocapsid proteins (NP) of NP.229E, NP.NL63, NP.OC43, and NP.HKU1, applying a graphical analysis to describe the relation between them.

We used Poisson regression with robust variance [6] to calculate unadjusted and adjusted incidence rate ratios and corresponding 95% as estimates of the association between WHO scores, which are considered as dichotomous variables (0 corresponding to 0–4 score and 1 corresponding to 5–8) and clinical statuses (i.e., NP or other antibodies). Adjusted ratios were obtained using gender and age as confounders. A *p*-value < 0.05 was considered as statistically significant”. Statistical analyses were performed using R version 4.0.3 (2020-10-10).

## 3. Results

Subjects were mainly females (52.6%, Table 1), younger than 65 years (50.0%), with no comorbidity (70.5%) and no opportunistic infection (56.4%). The majority of subjects was seronegative for seasonal coronaviruses (67.9% for anti-HCoV-299E, 67.9% for anti-HCov-NL63, and 84.6% for anti-HCoV-OC43 NP IgG). Moreover, 51.3% of subjects had no detectable SARS CoV-2 antibodies.

**Figure 1:**
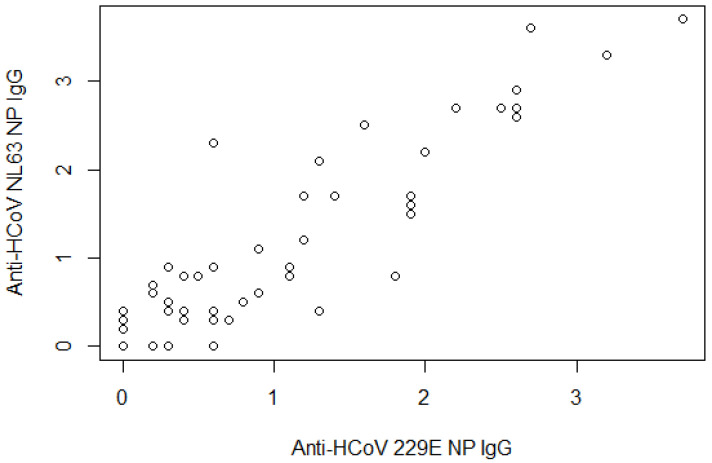
Scatterplot between anti-HCoV 229E NP and anti-HCoV NL63 NP IgG. The partial correlation coefficient was adjusted by age and sex = 0.903 (*p*-value < 0.0001).

**Figure 2:**
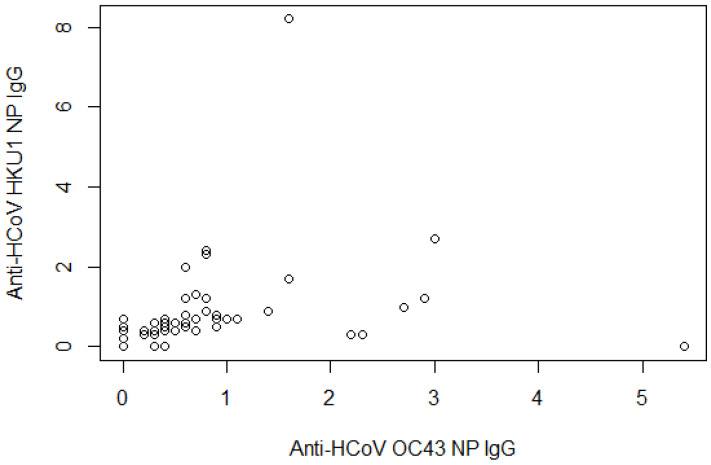
Scatterplot between anti-HCoV OC43 NP and anti-HCoV HKU1 NP IgG. The partial correlation coefficient was adjusted by age and sex = 0.306 (*p*-value < 0.006).

Anti-HCoV 229E NP and NL63 NP IgG were correlated (Figure 1, partial correlation coefficient = 0.903, *p*-value < 0.0001), as were anti-HCoV OC43 NP and HKU1 NP IgG (Figure 2, coefficient = 0.306, *p*-value < 0.006).

Table 2 summarizes the results of the logistical regression model, adjusted for comorbidities, sex, and age. Variables significantly associated with worse outcomes (WHO clinical severity ≥5) were co-infections (IRR: 2.75; 95% CI: 1.56, 4.83), anti-HCoV 229E nucleocapsid IgG (1.87; 1.22, 2.87), anti-HCoV NL63 nucleocapsid IgG (1.80; 1.18, 2.94), and the combination of the latter 2 IgGs (1.94; 1.27, 2.98). XXX

## 4. Discussion

The *Coronaviridae* family includes the 7 most known human coronaviruses that cause mild to moderate respiratory infections (i.e., HCoV-229E, HCoV-NL63, HCoV-OC43, HCoV-HKU1) as well as severe illness and death (MERS CoV, SARS CoV, SARS CoV-2) (summarized in Table 3). Severe infections induce hyperinflammatory responses that are often intensified by host adaptive immune pathways to profoundly advance disease severity. Proinflammatory responses are triggered by HCoV entry mediated by host cell surface receptors. Interestingly, 5 of the 7 strains use 3 cell surface metallopeptidases (CD13, CD26, and ACE2) as receptors, whereas the others employ O-acetylated-sialic acid (a key feature of metallopeptidases) for entry. It is unknown as to why HCoV evolved to use peptidases as their receptors, yet the peptidase activities of the receptors are dispensable, suggesting the virus uses/benefits from other functions of these molecules. Indeed, these receptors participate in the immune-modulatory pathways that contribute to the pathological hyperinflammatory response [7].

In this study, we have confirmed a previously recognized predictor of clinical outcomes (co-infections) and added previous immunity to alphacoronaviruses as an additional risk factor for WHO clinical severity score ≥ 5. There is little evidence of a correlation between SARS CoV-2 responses and HKU1 and NL63 responses [8]. No cross-reactivity of the SARS CoV-2 RBD-targeted antibodies was observed with HKU1, 229E, OC43, and NL63 [9]. In particular, antibodies against seasonal coronaviruses do not neutralize SARS-CoV-2 [10,11], with the only possible exception of 229E [12]. Nevertheless, one study reported cross-reactivity in anti-S2 antibodies between OC43 and SARS CoV-2 [13]. There is weak evidence of pre-existing SARS CoV-2 cross-reactive serum antibodies and limited cross-reactive memory B cells in pre-pandemic donors [14], and cross-reactivity to NL63 and 229E was more common in sub-Saharan Africa than in the USA [15]. Another study identified cross-reactivity between antibodies directed against SARS CoV-2 spike epitope 421–434 and NL63-RBM3 peptides [16]. Synchronous increase of OC43 IgG antibody levels was detected with SARS CoV-2 seroconversion in a subset of subjects for whom early infection sera were available before their SARS CoV-2 seroconversion, suggestive of an OC43 memory response triggered via SARS CoV-2 infection [17]. Among 17 severe COVID-19 cases, B-cell clones directed against seasonal CoV dominated and strongly increased over time. Seasonal CoV IgG responses that did not neutralize SARS CoV-2 were boosted well beyond detectable cross-reactivity during COVID19, particularly for an OC43 spike [18]. This was suggestive of OAS, which is theorized to have dismal consequences for coronaviruses [19,20]. ADE has been reported following vaccination or secondary infections with another coronavirus, RSV, Ebola, macrophage-tropic viruses (such as dengue virus), or non-macrophage-tropic respiratory viruses (such as RSV and measles). A detailed analysis has shown that antibodies to any viral epitope can induce ADE when present in sub-optimal titers or is of low affinity [21,22]. Neutralizing antibodies triggered by the sequential immunization of mice against SARS CoV and SARS CoV-2 are dominantly against the one that is used for priming [23]. Up to 50% of recovered SARS CoV-2 patients have been shown to mount antibody responses against unique epitopes of OC43, that were not detectable in unexposed individuals [24].

Complementary to our findings, patients with critical COVID-19 had significantly lower levels of OC43 and HKU1 nucleoprotein-specific antibodies compared to other COVID-19 patients [25]. The prognostic role of low OC43 antibodies was confirmed by another study: OC43 negative inpatients had an increased risk of critical disease (adjusted odds ratio 2.8), higher than the risk by increased age or body mass index (BMI), and lower than the risk by male sex [26]. These findings could also imply convalescent plasma collections (CCP): e.g., CCP units with greater NL63 antibody responses and lower HKU1 antibodies had higher neutralizing antibodies to the SARS CoV-2 receptor-binding domain (RBD) [27]. Another study found better outcome in recipients of CCP units with higher anti-NL63 or anti-OC43 antibodies [28].

Our study has several limitations, most importantly the low number of patients in several subgroups, which limits the statistical power and results in the wide confidence intervals of estimates reported in our study. We used a cross-sectional design and thus cannot exclude selection biases, particularly the incidence–prevalence one; however, we included consecutive patients without selection on disease severity. Given the current contradictory landscape, further studies on the prognostic role of previous immunity against endemic coronaviruses and prognosis of COVID-19 are warranted.

## Figures and Tables

**Table 1:** Distribution of demographic and clinical characteristics of the 78 subjects included in this study.

Category	No.	%
Gender		
Female	41	52.6
Male	37	47.4
Age, years		
<65	39	50.0
65–75	20	25.6
>75	19	24.4
Cardiological comorbidities		
No	55	70.5
Yes	17	21.8
NA	6	7.7
Infections		
No	44	56.4
Yes	25	32.1
NA	9	11.5
Anti-HCoV-229E NP IgG		
Mean readings ± SD	0.8	0.9
Negative	53	67.9
Positive	25	32.1
Anti-HCoV-NL63 NP IgG		
Mean readings ± SD	0.9	1.0
Negative	53	67.9
Positive	25	32.1
Anti-HCoV-229E NP IgG + anti-NL63 NP IgG		
None	49	62.8
One	8	10.3
Both	21	26.9
Anti-HCoV-229E NP IgG + anti-HCoV-NL63 NP IgG		
Negative	49	62.8
Positive	29	37.2
Anti-HCoV-OC43 NP IgG		
Mean readings ± SD	0.6	0.9
Negative	66	84.6
Positive	12	15.4
Anti-HCoV-HKU1 NP IgG		
Mean readings ± SD	0.6	1.1
Negative	67	85.9
Positive	11	14.1
Anti-HCoV-OC43 NP IgG + anti-HCoV-HKU1 NP IgG		
None	60	76.9
One	13	16.7
Both	5	6.4
Anti-HCoV-OC43 IgG+ Anti-HCoV-HKU1 IgG		
Negative	60	76.9
Positive	18	23.1
Anti-SARS-CoV-2 RBD IgG (Mikrogen), mean readings ± SD	2.7	3.2
Anti-SARS-CoV-2 S1 IgG (Mikrogen), mean readings ± SD	3.4	4.0
Cumulative number of anti-SARS-CoV-2 positive IgG specificities (Mikrogen)		
0	40	51.3
1	2	2.6
2	4	5.1
3	32	41.0
Final anti-SARS-CoV-2 serological diagnosis (Mikrogen)		
Negative	40	51.3
Positive	38	48.7
Anti-S1/S2 IgG (DiaSorin), mean readings ± SD	109.0	82.2
Anti-S1/S2 IgG (DiaSorin)		
Negative	6	7.7
Positive	24	30.8
NA	48	61.5

NA, not applicable; SD, standard deviation; NP nucleoprotein; RBD, receptor binding domain; S1/S2, Spike protein subunits S1 and S2.

**Table 2:** Crude and adjusted incidence rate ratio values and corresponding 95% confidence intervals (95% CIs) for disease gravity (World Health Organization (WHO) score ≥ 5 versus WHO score < 5) and selected clinical predictors.

	WHO Score < 5	WHO Score ≥ 5	IRR	(95% CI)	IRR Adjusted *	(95% CI)
Infections						
No	33 (82.5)	11 (37.9)	1	(ref.)	1	(ref.)
Yes	7 (17.5)	18 (62.1)	2.88	(1.63, 5.10)	2.75	(1.56, 4.83)
Anti-HCoV 229E NP IgG						
No	34 (81.0)	19 (52.8)	1	(ref.)	1	(ref.)
Yes	8 (19.0)	17 (47.2)	1.90	(1.21, 2.98)	1.87	(1.22, 2.87)
Anti-HCoV NL63 NP IgG						
No	33 (78.6)	20 (55.6)	1	(ref.)	1	(ref.)
Yes	9 (21.4)	16 (44.4)	1.70	(1.07, 2.68)	1.80	(1.18, 2.74)
Anti-HCoV 229E NP + Anti-HCoV NL63 NP IgG						
None	31 (73,8)	18 (50.0)	1	(ref.)	1	(ref.)
One	5 (11.9)	3 (8.3)	1.02	(0.39, 2.70)	1.34	(0.49, 3.63)
Both	6 (14.3)	15 (41.7)	1.94	(1.23, 3.08)	1.94	(1.27, 2.98)
Anti-HCoV 229E NP + Anti-HCoV NL63 NP IgG						
None	31 (73.8)	18 (50.0)	1	(ref.)	1	(ref.)
At least one	11 (26.2)	18 (50.0)	1.69	(1.06, 2.70)	1.82	(1.17, 2.81)
Anti-HCoV OC43 NP IgG						
No	36 (85.7)	30 (83.3)	1	(ref.)	1	(ref.)
Yes	6 (14.3)	6 (16.7)	1.10	(0.59, 2.06)	0.97	(0.53, 1.79)
Anti-HCoV HKU1 NP IgG						
No	37 (88.1)	30 (83.3)	1	(ref.)	1	(ref.)
Yes	5 (11.9)	6 (16.7)	1.22	(0.66, 2.23)	1.05	(0.54, 2.03)
Anti-HCoV OC43 NP IgG + Anti-HCoV HKU1 NP IgG						
None	34 (81.0)	26 (72.2)	1	(ref.)	1	(ref.)
One	5 (11.9)	8 (22.2)	1.42	(0.84, 2.39)	1.34	(0.80, 2.25)
Both	3 (7.1)	2 (5.6)	0.92	(0.30, 2.83)	0.74	(0.24, 2.31)
Anti-HCoV-OC43 NP IgG + anti-HCoV-HKU1 NP IgG						
None	34 (81.0)	26 (72.2)	1	(ref.)	1	(ref.)
At least one	8 (19.0)	10 (27.8)	1.28	(0.77, 2.13)	1.16	(0.69, 1.94)
Number of positive IgGs against HCoV						
0	27 (64.3)	16 (44.4)	1	(ref.)	1	(ref.)
1	7 (16.7)	3 (8.3)	0.81	(0.29, 2.26)	0.92	(0.36, 2.38)
2	5 (11.9)	10 (27.8)	1.79	(1.05, 3.04)	1.78	(1.05, 3.03)
≥3	3 (7.2)	7 (19.5)	1.88	(1.07, 3.31)	1.76	(1.02, 3.04)
Number of positive IgGs against HCoV						
0	27 (64.3)	16 (44.4)	1	(ref.)	1	(ref.)
>0	15 (35.7)	20 (55.6)	1.54	(0.94, 2.50)	1.56	(0.99, 2.47)
Number of positive IgG specificities against SARS CoV-2						
0	27 (64.3)	13 (36.1)	1	(ref.)	1	(ref.)
1–2	5 (11.9)	1 (2.8)	0.51	(0.08, 3.28)	0.47	(0.08, 2.67)
3	10 (23.8)	22 (61.1)	2.11	(1.27, 3.51)	1.91	(1.14, 3.18)
Number of positive IgG specificities against SARS CoV-2						
0	27 (64.3)	13 (36.1)	1	(ref.)	1	(ref.)
>0	15 (35.7)	23 (63.9)	1.86	(1.11, 3.13)	1.65	(0.98, 2.79)
Final serological SARS-CoV-2 diagnosis (Mikrogen)						
No	27 (64.3)	13 (36.1)	1	(ref.)	1	(ref.)
Yes	15 (35.7)	23 (63.9)	1.86	(1.11, 3.13)	1.65	(0.98, 2.79)
Final serological SARS-CoV-2 diagnosis (DiaSorin)						
No	5 (27.8)	1 (8.3)	1	(ref.)	1	(ref.)
Yes	13 (72.2)	11 (91.7)	2.75	(0.42, 17.89)	2.49	(0.39, 15.93)

Incidence rate ratio (IRR) were calculated using Poisson regression model with robust variance * adjusted for sex and age.

**Table 3:** Key features of HCoVs affecting humans (modified from ref [7]).

Genus	Species	Cellular Receptor	Sequence Identity to SARS CoV-2
Alpha	NL63	ACE2	49%
	229E	Aminopeptidase N	48%
Beta	SARS CoV-2	ACE2	100%
	SARS CoV		80%
	MERS CoV	DPP-IV	54%
	HKU-1	sialoglycan-based receptors with 9-O-acetylated sialic acid (9-O-Ac-Sia)	52%
	OC43		51%

## Data Availability

The data presented in this study are available on request from the corresponding author.
